# The Micro–Macro Interlaminar Properties of Continuous Carbon Fiber-Reinforced Polyphenylene Sulfide Laminates Made by Thermocompression to Simulate the Consolidation Process in FDM

**DOI:** 10.3390/polym14020301

**Published:** 2022-01-12

**Authors:** Jiale Hu, Suhail Mubarak, Kunrong Li, Xu Huang, Weidong Huang, Dongxian Zhuo, Yonggui Li, Lixin Wu, Jianlei Wang

**Affiliations:** 1 School of Chemistry, Fuzhou University, Fuzhou 350116, China; hujiale@fjirsm.ac.cn (J.H.); likunrong@fjirsm.ac.cn (K.L.); 2 Fujian Key Laboratory of Novel Functional Textile Fibers and Materials, Minjiang University, Fuzhou 350108, China; 3 CAS Key Laboratory of Design and Assembly of Functional Nanostructures, Fujian Key Laboratory of Nanomaterials, Fujian Institute of Research on the Structure of Matter, Chinese Academy of Sciences, Fuzhou 350002, China; 4 Department of Nano Electronics Materials and Sensors, Institute of Electronics and Communication Engineering, Saveetha School of Engineering, Saveetha Institute of Medical and Technical Sciences, Chennai 602105, Tamil Nadu, India; suhailmubarak@gmail.com; 5 School of Mechanical & Automotive Engineering, Fujian University of Technology, Fuzhou 350118, China; huangxu@fjut.edu.cn (X.H.); hwd@fjut.edu.cn (W.H.); 6 College of Chemical Engineering and Materials Science, Quanzhou Normal University, Quanzhou 362000, China; dxzhuo@qztc.edu.cn; 7 Engineering Research Center of Polymer Green Recycling of Ministry of Education, Fujian Normal University, Fuzhou 350007, China

**Keywords:** continuous fiber-reinforced composites, mechanical properties, interlaminar properties, prepreg, 3D printing simulation

## Abstract

Three-dimensional (3D) printing of continuous fiber-reinforced composites has been developed in recent decades as an alternative means to handle complex structures with excellent design flexibility and without mold forming. Although 3D printing has been increasingly used in the manufacturing industry, there is still room for the development of theories about how the process parameters affect microstructural properties to meet the mechanical requirements of the printed parts. In this paper, we investigated continuous carbon fiber-reinforced polyphenylene sulfide (CCF/PPS) as feedstock for fused deposition modeling (FDM) simulated by thermocompression. This study revealed that the samples manufactured using a layer-by-layer process have a high tensile strength up to 2041.29 MPa, which is improved by 68.8% compared with those prepared by the once-stacked method. Moreover, the mechanical–microstructure characterization relationships indicated that the compactness of the laminates is higher when the stacked CCF/PPS are separated, which can be explained based on both the void formation and the nanoindentation results. These reinforcements confirm the potential of remodeling the layer-up methods for the development of high-performance carbon fiber-reinforced thermoplastics. This study is of great significance to the improvement of the FDM process and opens broad prospects for the aerospace industry and continuous fiber-reinforced polymer matrix materials.

## 1. Introduction

Carbon fiber-reinforced thermoplastic (CFRTP) offers significant advantages thanks to the excellent mechanical properties, high specific stiffness, high energy absorption, thermal stability, high fatigue limit, and long service life [1,2,3], which are widely used in biomedical, aerospace, automotive, and industrial fields [4,5]. Additive manufacturing, also known as three-dimensional (3D) printing, of continuous fiber-reinforced composites has been developed in recent years as an alternative means to manufacture complex structures [6,7,8], as well as custom products, factory tools, and parts [9]. Fused deposition modeling (FDM) is the most widespread 3D printing technology due to its simple process, relatively low cost, and great design flexibility [10,11]. Continuous fiber-reinforced composites have attracted increasing attention since 2014 when MarkForged^®^ (Watertown, NY, USA) developed the first continuous fiber printer [12]. Dickson et al. [13] proved that the tensile and flexural properties of fiber-reinforced nylon are 5 to 6.3 times higher than those of pure nylon, while the enhancement of mechanical properties of materials reinforced by short fiber is quite limited [14,15]. The previous studies suggested that the FDM process may lead to the formation of a porous structure in the CFRTP parts due to insufficient thermo-mechanical consolidation of the material, which may lead to poor mechanical strength [16,17,18,19]. The mechanical properties of the material largely depend on the bonding quality of the beads during the print head extrusion and the molecular diffusion of the polymer chain to the contact surface [20,21]. The main factor affecting the mechanical properties of FDM composites is the poor interlaminar adhesion between fiber and matrix due to the smoothness, non-polarity, low knot, and chemical inertia of carbon fiber (CF) [21,22,23]. Therefore, the interlaminar bonding performance is of great significance in FDM-printed, fiber-reinforced composite parts. Although extensive investigations have been carried out on the printing conditions and the properties of the FDM-printed parts [24], no thorough research exists regarding the influence of the process parameters on the microstructures.

To compensate for the limitations mentioned above and maximize the fiber–matrix interlaminar adhesion and thermomechanical consolidation properties, we employed the hot-pressing molding technique to simulate the process characteristics of the “in situ consolidation” of the FDM 3D printing process due to the similar thermal cycles and interfacial behaviors in both processes. The microstructures and the process-induced defects of continuous carbon fiber-reinforced polyphenylene sulfide (CCF/PPS) composites were studied, and the effects of the microstructure on the mechanical properties were evaluated. The effect of the interlaminar stacking sequence of continuous CFRTP on the mechanical properties was investigated. The macro-mechanical response and failure mechanism were analyzed by using scanning electron microscopy (SEM) to further study the performance of the CCF/PPS laminates, proving that the changes in the microstructure of the CCF/PPS composites would directly affect its macro-mechanical performance.

## 2. Experimental Section

Three different CCF/PPS laminates with a variation of layer-up methods were prepared via thermocompression to simulate the process characteristics of the “in situ consolidation” of the FDM 3D fiber printing process (see the Supplementary Materials: S1. Process of preparation, Figure S1). For convenience, the CCF/PPS laminates prepared via layer-by-layer stacking and once-stacking were denoted by C13 or S13 in the following, respectively. The number 13 means that they were all pressed 13 times, while S1 was pressed only once. As shown in Table 1, the single lamina of C13 was 0.17 mm in thickness, slightly lower than that of the S13. In addition, S5, C5, and S1_L5_ were also prepared using the same method, as the 13-layer composites were too thick for the tensile testing. All the samples were cut into the corresponding testing specimens, and the layer height is shown in Table 1. The dimensions of the specimens were 67 mm (length) × 8 mm (width) × ~1 mm (thickness) for the tensile test and 16 mm (length) × 4.8 mm (width) × ~2.4 mm (thickness) for the ILSS test. The schemas of laminate preparation and test specimens are presented in Supplementary Figures S2, S4 and S5, respectively.

The interlaminar properties of the laminates were evaluated via nanoindentation using the nanoindenter instrument (Tribo Indenter 750, Hysiteon Inc., Minneapolis, MN, USA) (see the Supplementary Materials: S2. Nanoindentation). The surface porosity was determined via microscopy image analysis using an optical microscope (Leica Z16 APO, Leica Microsystems, Wetzlar, Germany), and the volume porosity percentage was estimated using density measurement (see the Supplementary Materials: S3. Calculation of volume porosity percentage). The tensile and interlaminar shear strength (ILSS) tests were performed using the universal testing machine (AGX-100 plus, Shimadzu, Kyoto, Japan) according to ASTM D3039 and ASTM D2344 standards. The typical fracture surfaces produced via the ILSS test were observed using a scanning electron microscope (SEM) (JSM5200, JEOL, Tokyo, Japan). The storage modulus was performed using a dynamic mechanical analyzer (DMA) (Q800, TA, New Castle, DE, USA) under a single cantilever beam mode at the heating rate of 5 °C/min. The electrical conductivity was performed using a four-probe scheme. The thermal conductivity was performed using the transient hot-wire technique. More details of the experimental section are attached in the Supplementary Materials.

## 3. Results and Discussion

### 3.1. Interlaminar Properties and Microstructures

**Figure 1:**
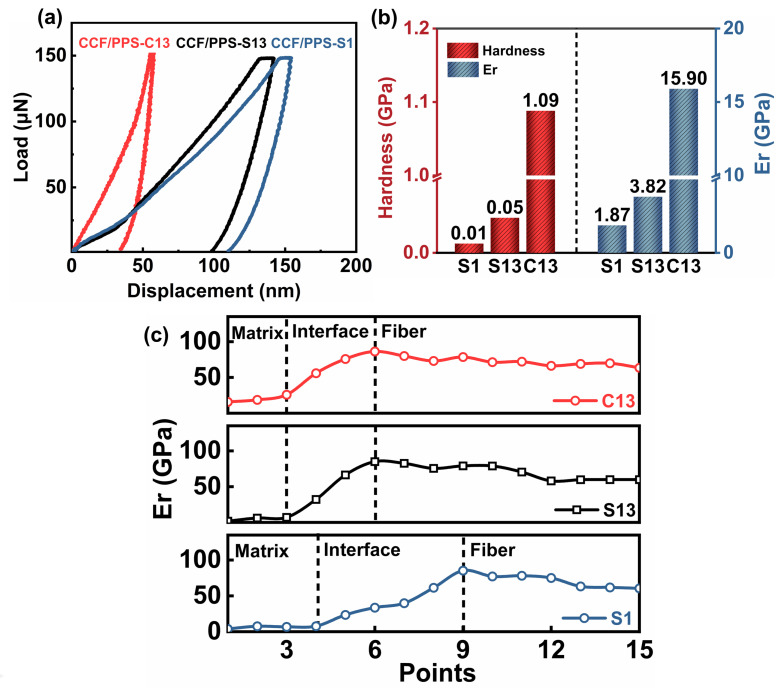
(**a**) Nanoindentation load–displacement curves of the composites at loads of 150 mN; (**b**) nanohardness and elastic modulus (*E*_r_) values of different composites; (**c**) modulus profiles for different composites.

The micro interlaminar properties of the laminates were investigated using nanoindentation tests [25,26]. About 15 indentations were performed on each selected area, and each indentation was programmed to apply a maximum force of 150 μN on the polished surface at a loading and unloading rate set to 150 μN /min. Each probe test was first performed on the PPS resin matrix and then to the carbon fiber region from left to right. The method described by Oliver-Pharr was followed to characterize the elastic modulus (*E*_r_) and hardness [27]. Figure 1a illustrates the representative nanoindentation load–displacement curves, from which the *E*_r_ and hardness of the composites can be derived (see the Supplementary Materials: S2. Nanoindentation). As shown in Figure 1b, the *E*_r_ values for C13, S13, and S1 composites are 15.9 GPa, 3.82 GPa, and 1.87 GPa, respectively. The samples prepared via layer-by-layer stacking (C13) exhibit higher values of *E*_r_ and hardness compared to those of once-stacked (S13 and S1), which may be related to interlaminar bonding performance. According to Figure 1c, the typical 15 indentations were chosen, and the fiber, matrix, and their interphase are distinguishable in the modulus results. It was evident that the modulus of the carbon fiber is much higher than that of the PPS matrix and that the intermediate region is present in the fiber–matrix interface. The interface transition region exhibits an increasing trend in both hardness and modulus from matrix to fiber. The resistance of the hybrids to indentation presented a trend of slow decrease, with the decrease in *E*_r_ due to the plasticizing effect, which is consistent with previous studies [28]. It is also evident that the layer-by-layer method disperses the interlaminar excess energy when the nanocomposites tend to gather around the surface of the fiber, leading to better interlaminar bonding, which can also be determined by subsequent experiments.

**Figure 2:**
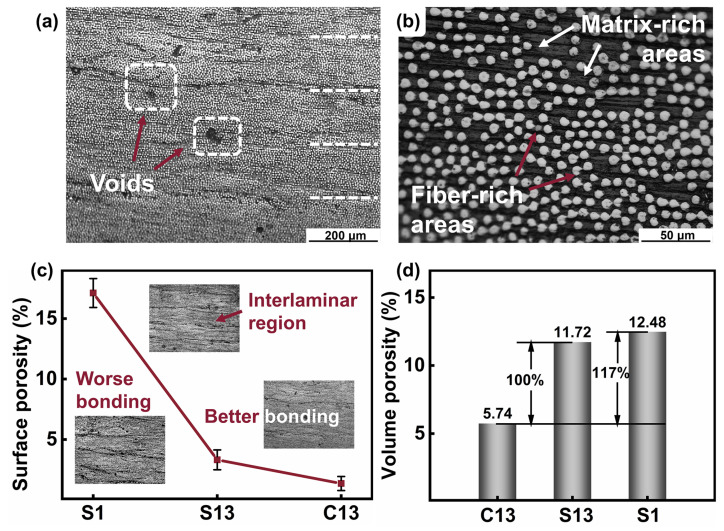
Cross-section of the CCF/PPS-C13 sample at different magnification levels and porosity measurements: (**a**) interlaminar region (horizontal dashed lines) and voids; (**b**) alternated matrix-rich and fiber-rich regions; (**c**) surface porosity percentage; and (**d**) volume porosity percentage.

The microscopy images—taken at different magnification levels—of the cross-section of the unidirectional C13 sample were used to determine the surface porosity percentage and thus comprehensively evaluate the microstructures and the process-induced defects. It has been extensively documented that image analysis of material porosity is in good agreement with actual porosity percentage [29]. As shown in Figure 2a,b, it is evident that there are two typical voids (interlaminar and intralaminar), and most of them occur in matrix-rich areas where the interlaminar regions (horizontal dashed lines in Figure 2a) are visible after the process. The more specific micrographs of the CCF/PPS composite with corresponding layer-up methods are presented in Supplementary Figure S7. It might demonstrate that the fiber distribution is inhomogeneous and the molecular diffusion of the matrix is uncharacterized, which can distinguish the large matrix-dominated from the high fiber density area. According to Figure 2c, a decrease in the surface porosity can be observed, which is due to the different layer-up methods and frequency of pressing. The surface porosity of the C13 sample is about 1.37%, far lower than the S1 sample (17.12%). For the different layer-up types, the C13 sample has lower surface porosity than the S13 sample. For the different frequency of pressing, the S13 sample has lower porosity than the S1 sample. Such differences are shown to alter the interlaminar properties of the composites. However, the surface porosity has strong stochasticity due to the randomly chosen cross-section.

To obtain more accurate conclusions, the volume porosity values following the density method measurements are also presented in Figure 2d, which are roughly consistent with that of the surface porosity. The volume porosity of the C13 sample is about 5.74%, only half that of the S13 sample (11.77%), and significantly lower than the S1 sample. For the samples with the lowest porosity, the C13 composite has an indentation hardness of 1.088 GPa and *E*_r_ of 15.9 MPa, far more than other samples (Figure 1c). The S1 has the highest volume porosity of 17.1% due to the lowest number of pressings and the once-stacked method. This illustrates that the structure of CCF/PPS composites prepared via lamination is more compact and smoother than those prepared using the once-stacked method, thus developing better adhesion and fusion of the layers and improving the mechanical performance. However, the frequency of pressing does not have a significant effect on the volume porosity. This might be due to a couple of reasons, the foremost being that the frequency of pressing did not enhance fiber–matrix interlaminar adhesion enough to affect the diffusion of molecules.

### 3.2. Mechanical Performance and Other Properties

**Figure 3:**
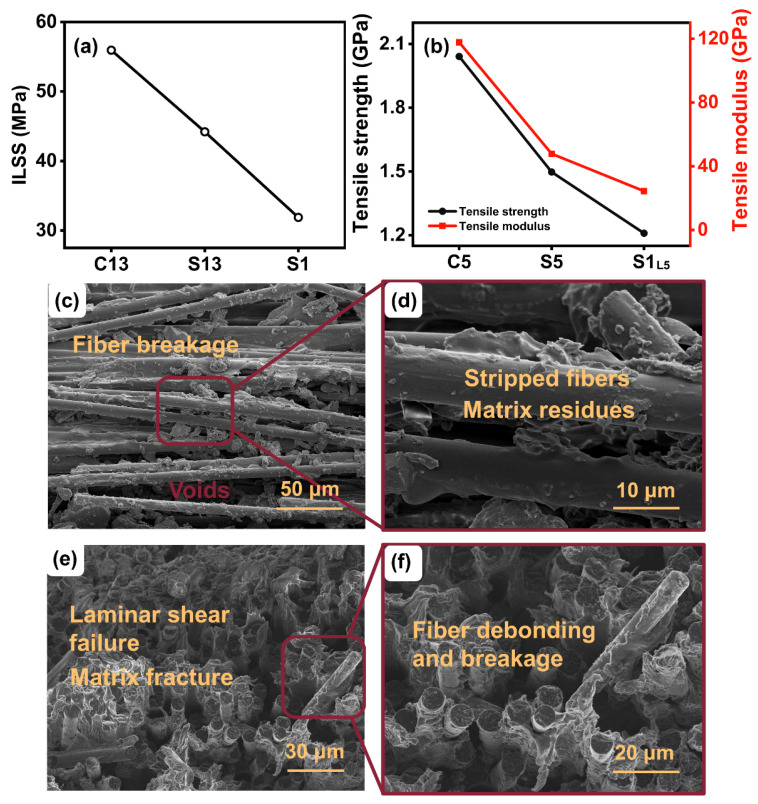
(**a**) ILSS of different composites; (**b**) tensile properties; and SEM images of the fracture surface: (**c**) fiber breakage zones and voids; (**d**) stripped fibers with little impregnated resin; (**e**) laminar shear failure and matrix fracture zones; and (**f**) fiber debonding zones.

In addition to the micro interlaminar properties, the macro interlaminar behaviors of the laminates were further investigated via mechanical testing. The average measured mechanical values of the CCF/PPS composites prepared using both methods are shown in Figure 3. As seen in Figure 3a, the ILSS of the laminates prepared using layer-by-layer stacking yields a higher strength than those prepared using the once-stacked method, as expected, which is 55.94, 44.20, and 31.89 MPa for C13, S13, and S1 composites, respectively. The maximum increment of about 75.4% in ILSS can be observed for the composites fabricated via layer-by-layer (C13), which is likely due to the lamination failure which is dependent on the interlaminar bonding interactions between two adjacent layers. Tensile tests were conducted on the thinner samples since the 13-layer samples were too thick for testing. As shown in Figure 3b, the tensile strengths of C5, S5, and S1_L5_ are 2041.29, 1497.52, and 1209.29 MPa, respectively. For the CCF/PPS laminates prepared using the layer-by-layer stacking method (C5), the tensile strengths are significantly higher (about 68.8%) than those prepared using the once-stacked method (S5 and S1_L5_); for the laminates with the same number of layers (S5 and S1_L5_), the frequency of pressing did not affect the tensile strengths much. The same for the tensile modulus: the CCF/PPS laminates prepared using the layer-by-layer stacking method (C5) display a higher tensile modulus than those prepared using the layer-up method. In terms of damage morphology, the fiber breakage, fiber–matrix debonding, and the laminar shear failure can be observed in the obtained SEM images of the fracture surface caused by the ILSS test (Figure 3c–f). From these, we can make the conclusion that the main damage morphology of the laminates is more likely to prefer fiber–matrix debonding. The pulled-out fibers with matrix residues indicate the proper interlaminar adhesion between the fibers and the matrix. It can be observed that there are obvious pores between these continuous fibers (Figure 3d), which may hinder the fluidity of the PPS matrix and lead to the incomplete impregnation of the CCF/PPS composites. 

**Figure 4:**
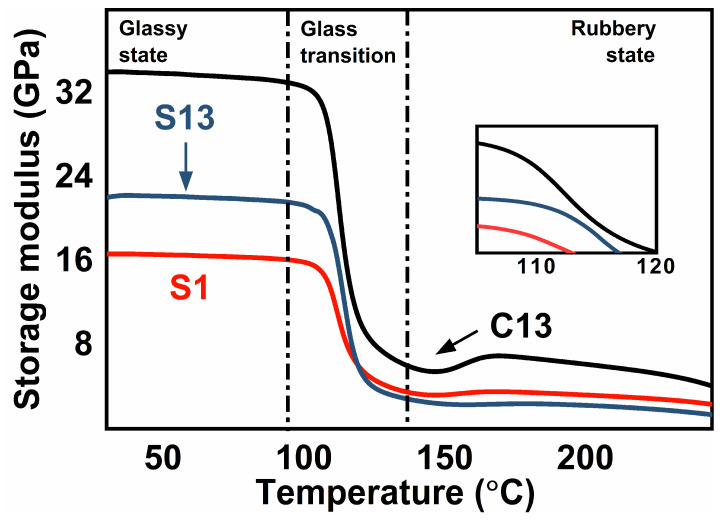
The DMA plots of storage modulus under different temperatures.

For the DMA tests, the layer-by-layer stacking method also induced a higher storage modulus of the CCF/PPS hybrid laminates at the rubbery state. Figure 4 shows the variations of storage modulus as a function of temperature. As one may expect, a significant enhancement in the storage modulus of C13 composite can be observed, leading to a more successful binding interface. Moreover, the increased pressure times result in higher storage modulus, also due to better bonding between the layers of the laminates. Likewise, the electrical conductivity and thermal conductivity of the CCF/PPS composites manufactured using the layer-by-layer stacking method (C13) were higher than those using the once-stacked method (S13 and S1). This implied that the higher compactness and lower porosity were beneficial to the establishment of efficient continuous conductive pathways. More details are attached in the Supplementary Materials (S4. the electrical and thermal performance).

Overall, we successfully employed the hot-pressing molding technique to simulate an FDM 3D printing technique due to the similar thermal cycles and interfacial behaviors in both processes. It can be concluded that the layer-up methods affect the interlaminar bonding force between fiber and matrix and layers of the CCF/PPS composites by altering their microstructures. That is, the CCF/PPS composites prepared using the layer-by-layer stacking method display a better adhesion and fusion of the layers, and the better micro-mechanical properties of the CCF/PPS laminates are attributed to the better dispersion and stronger interlaminar bonding force between fiber and matrix. These results might provide useful insights into the FDM printing of high-performance continuous carbon fiber-reinforced composites and could be extensively used in aerospace, automotive, and industrial fields.

## 4. Conclusions

In summary, continuous carbon fiber-reinforced PPS resin with different stacking sequences was successfully prepared, and the influence of the printing parameters on the micro–macro interlaminar properties of the laminates was evaluated. It was shown that micro-mechanical properties were proved to affect the macro-mechanical properties as well as thermal and electrical performance. The mechanical properties of the laminates were higher when the stacked CCF/PPS were separated, and composites with highly dispersed and highly oriented fibers can be manufactured, which were broadly consistent with the microstructural results. It is noticeable from these results that changing the layer-up methods could clearly minimize void formation and fiber breakage during the printing process, which induced a higher thermomechanical consolidation, higher compactness, and lower defects in the laminates. This study focused on the idea that the increase in porosity negatively affects the mechanical strength of the laminates by creating stress concentration points as well as reducing the fiber–matrix interlaminar adhesion, which has a certain reference value for perfecting the theoretical system and improving the 3D printing technology based on FDM.

## Figures and Tables

**Table 1:** Labels and thickness of the CCF/PPS composite laminates with corresponding layer-up methods and frequency of pressing.

Sample Label	Layer-Up Types	Frequency of Pressing	Thickness of Each Layer
C13	Layer-by-layer	13	0.17 mm
S13	Once-stacked	13	0.18 mm
S1	Once-stacked	1	0.21 mm
C5	Layer-by-layer	5	0.20 mm
S5	Once-stacked	5	0.21 mm
S1_L5_^a^	Once-stacked	1	0.23 mm

^a^ S1_L5_ is composed of 5 layers, prepared via once-stacked and pressed once methods.

## Data Availability

The authors confirm that the data supporting the findings of this study are available within the article.

## Supplementary Materials

The following supporting information can be downloaded at: https://www.mdpi.com/article/10.3390/polym14020301/s1, Figure S1: The schema of laminate preparation and test; Figure S2: The temperature and pressure of the hot compression molding process; Figure S3: The schema of the correlation between hot pressing technology and FDM 3D printing technology; Figure S4: The schema of laminate preparation; Figure S5: The tensile test specimen (a) and the ILSS test specimen (b); Figure S6: The electrical conductivity and thermal conductivity of different fiber reinforced composites manufactured by different layer-up methods; Figure S7: (a–c) The micrographs of the S1, S13 and C13 samples (from left to right); (d–f) the SEM images of the S1, S13 and C13 samples (from left to right).
