# Supplementary material for: The Micro–Macro Interlaminar Properties of Continuous Carbon Fiber-Reinforced Polyphenylene Sulfide Laminates Made by Thermocompression to Simulate the Consolidation Process in FDM
Source: Polymers (Basel). 2022 Jan 12;14(2):301. doi: 10.3390/polym14020301 (PMC8781787; doi:10.3390/polym14020301)
Supplement: Supplementary file 1 [file polymers-14-00301-s001.zip › polymers-1497282-supplementary.pdf]

The Micro–Macro Interlaminar Properties of Continuous Carbon Fiber-Reinforced Polyphenylene Sulfide Laminates Made by Thermocompression to Simulate the Consolidation Process in FDM

Jiale Hu [1,2,3], Suhail Mubarak [4], Kunrong Li [1,3], Xu Huang [5], Weidong Huang [5], Dongxian Zhuo [6], Yonggui Li [2,*], Lixin Wu [3,*] and Jianlei Wang [2,3,7,*]

[1] School of Chemistry, Fuzhou University, Fuzhou, Fujian 350116, P. R. China

[2] Fujian Key Laboratory of Novel Functional Textile Fibers and Materials, Minjiang University, Fuzhou, Fujian, 350108, China

[3] CAS Key Laboratory of Design and Assembly of Functional Nanostructures, Fujian Key Laboratory of Nanomaterials, Fujian Institute of Research on the Structure of Matter, Chinese Academy of Sciences, Fuzhou, Fujian 350002, P. R. China

[4] Department of Nano Electronics Materials and Sensors, Institute of Electronics and Communication Engineering, Saveetha School of Engineering, Saveetha Institutes of Medical and Technical Sciences, Chennai-602105, Tamil Nadu, India

[5] School of Mechanical & Automotive Engineering, Fujian University of Technology, Fuzhou, 350118, China

[6] College of Chemical Engineering and Materials Science, Quanzhou Normal University, Quanzhou 362000, China

[7] Engineering Research Center of Polymer Green Recycling of Ministry of Education, Fujian Normal University, Fuzhou 350007, China

[*] Correspondence: ygwxjd@sina.com (Y.L.); lxwu@fjirsm.ac.cn (L.W.); jlwang@fjirsm.ac.cn (J.W.)

Supporting Information

S1. Process of preparation

Three different CCF/PPS samples with variation layer-up methods were prepared to simulate the process characteristics of "in-situ consolidation" of FDM 3D fiber printing process with the procedure performed at temperature 310 ℃ and pressure 3 MPa. The schemas of laminate preparation are shown in Figure S1, Figure S2 and Figure S3. The polyphenylene sulfide (PPS) prepreg (TC1100 High Strength UD Tape, Carbon AS4A 221gsm, 34% RC) used in this study was 6.35 mm in width, with a fiber volume fraction of 50% and a tow size of 1K. The resultant continuous carbon fiber reinforced polyphenylene sulfide (CCF/PPS) laminates with different layer-up types and frequency of press were consolidated.

Three different carbon fiber reinforced thermoplastic (CFRTP) samples with variation layer-up methods were prepared. The first one was stacked and hot-pressed layer by layer. Each time the spline was stacked, pressure was applied, and so on until 13 layers, named C13. The second layer-up method was once-stacked. The laminates were properly stacked into 13 plies and hot-pressed once, named S1. Then, S1 was melted and pressed again under the same conditions, and so on until 13 times, named S13. The schema of the specimen preparation is shown in Figure S4. In addition, S5, C5, $S1_{5L}$ were prepared in the same method respectively, for that the 13-layer composites were too thick for the tensile testing. All the samples were cut into the corresponding testing specimens and the schema of the tensile test specimen and the ILSS test specimen were shown in Figure S5.

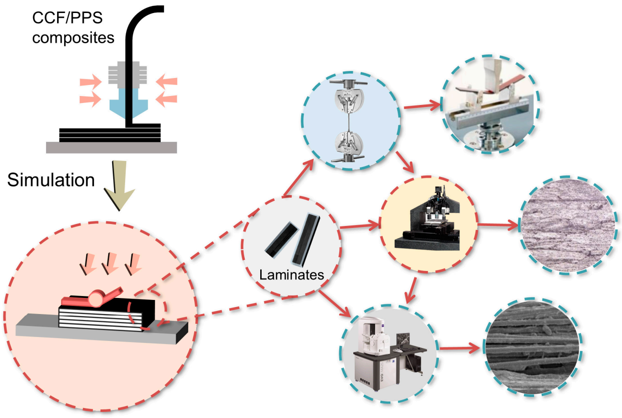

Figure S1. The schema of laminate preparation and test.

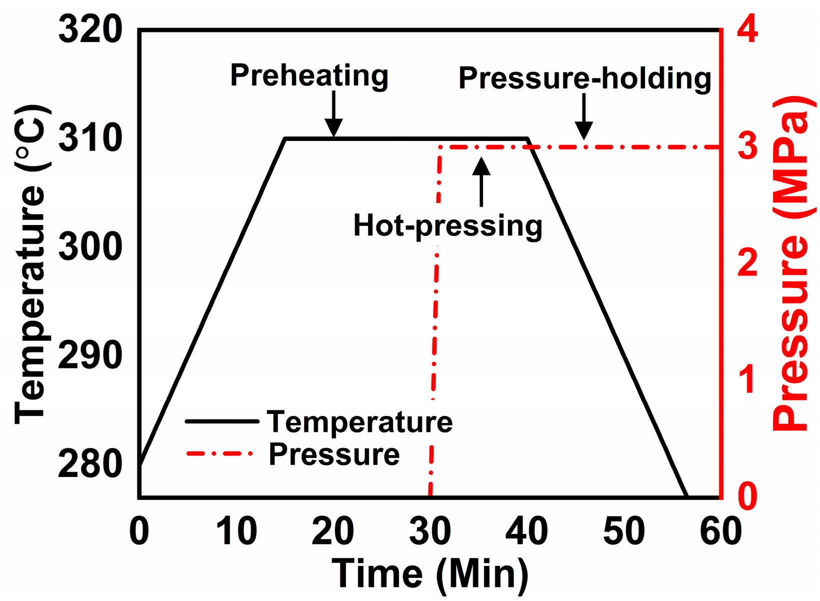

Figure S2. The temperature and pressure of the hot compression molding process.

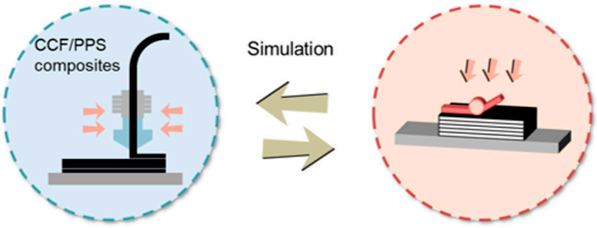

Figure S3. The schema of the correlation between hot pressing technology and FDM 3D printing technology.

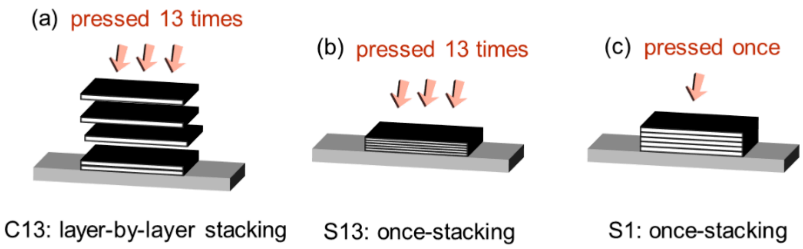

Figure S4. The schema of laminate preparation.

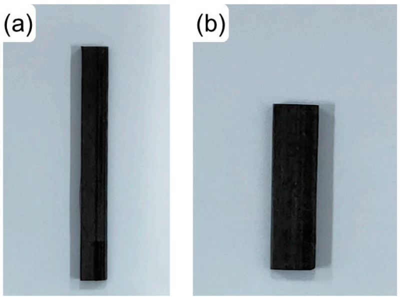

Figure S5. The tensile test specimen(a) and the ILSS test specimen(b).

S2. Nanoindentation

Nanoindentation was performed at a peak load (P_{max}) of 150 μN, which was based on the interphase thickness and the size of the specimen. The size of the indenter was 50 nm. The load parameter displayed the force for the currently selected point and the displacement parameter displayed the displacement for the currently selected points. The elastic modulus (E_r) and hardness of the CCF/PPS composites are determined according to the method described by Oliver-Pharr, calculated using equations (S1) and (S2). The calculation of the E_r is related to the initial slope of the unloading curve.

$$H = \frac{P_{max}}{A_c} \quad \text{(S1)}$$

$$S = \frac{dP}{dH} = \frac{2E_r\sqrt{A_c}}{\sqrt{\pi}} \quad \text{(S2)}$$

where P_{max} denotes the maximum applied load, A_c ($A_c = 24.56\ h_c^2$) denotes the projected contact area calculated at the depth of indentation (H), S and E_r are the contact stiffness and reduced elastic modulus, respectively. The E_r can be calculated from the following E_q (S3).

$$\frac{1}{E_r} = \frac{1-v_s^2}{E_s} \quad \text{(S3)}$$

where E_r denotes Young's of the sample and v_s denotes Poisson's ratio of the sample.

S3. Calculation of volume porosity percentage

Several cross sections were selected and polished by optical microscope (LeicaZ16APO), then the polished cross sections were observed under a microscope with a certain number of checkered reference objects, and finally the pores falling on the grid intersection are counted. ImageJ software was used to analyze the photos, and the porosity was obtained by statistical analysis of porosity from the point of view of image processing.

The volume porosity was measured by density measurement. The density of carbon fiber (CF), PPS, CCF/PPS composites and the mass fraction of polymer matrix in the composites were measured, and then the porosity can be determined by the formula.

$$V = 1 - \rho_m(w_r/\rho_r + w_s/\rho_s) \quad \text{(S4)}$$

where V is the volume porosity of the composites; ρ_m is the density of the composites; ρ_r the density of the PPS; ρ_s is the density of the CCF; w_r is the mass fraction of the PPS; w_s is the mass fraction of the CF.

S4. The electrical and thermal performance

The electrical conductivity and thermal conductivity of the CCF/PPS laminates were further characterized by four-probe scheme and transient hot-wire technique, respectively. It can be seen from Figure S3 that the electrical conductivity of the CCF/PPS composites manufactured by layer-by-layer stacking method (C13) reaches up to 0.3684 S cm^{-1}, displaying a significant increase of around nine-fold compared with that of prepared by the once-stacked method. It might be partially explained by the denser microstructure of the CCF/PPS composites prepared by layer-by-layer stacking method, resulting in a more efficient continuous conductive path. Likewise, the thermal conductivity of the CCF/PPS composites manufactured by layer-by-layer stacking method (C13) were higher than that prepared by once-stacked method (S13 and S1). This implicated that the higher compactness and lower porosity were beneficial to the establishment of efficient thermally conductive pathways.

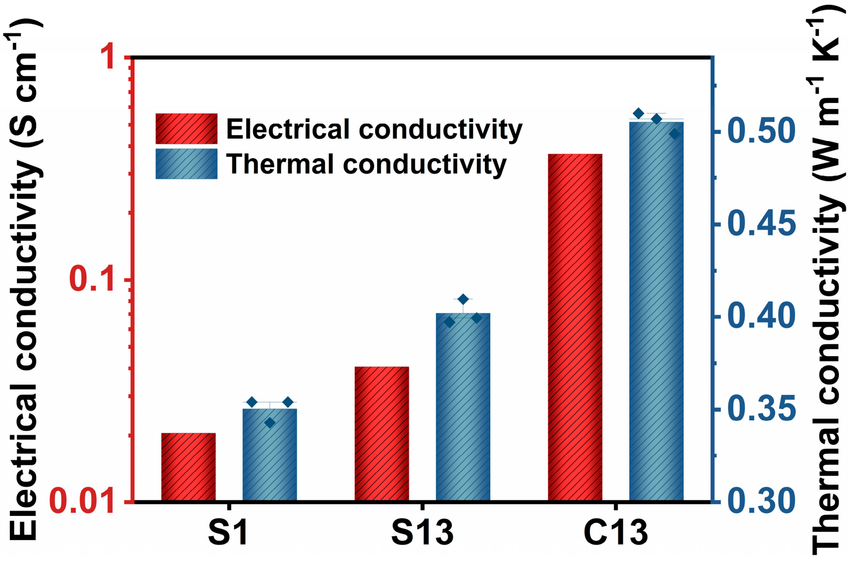

Figure S6. The electrical conductivity and thermal conductivity of different fiber reinforced composites manufactured by different layer-up methods.

S5. The microstructure

The micrographs of the CCF/PPS composite with corresponding layer-up methods were shown in Figure S7. It can be observed that the C13 sample has lower porosity than the S1 and S13 sample. Such differences were shown to alter the interlaminar properties of the composite, illustrating that the structure of CCF/PPS composites prepared by lamination was more compact and smoother than prepared once-stacked, thus developing better adhesion and fusion of the layers.

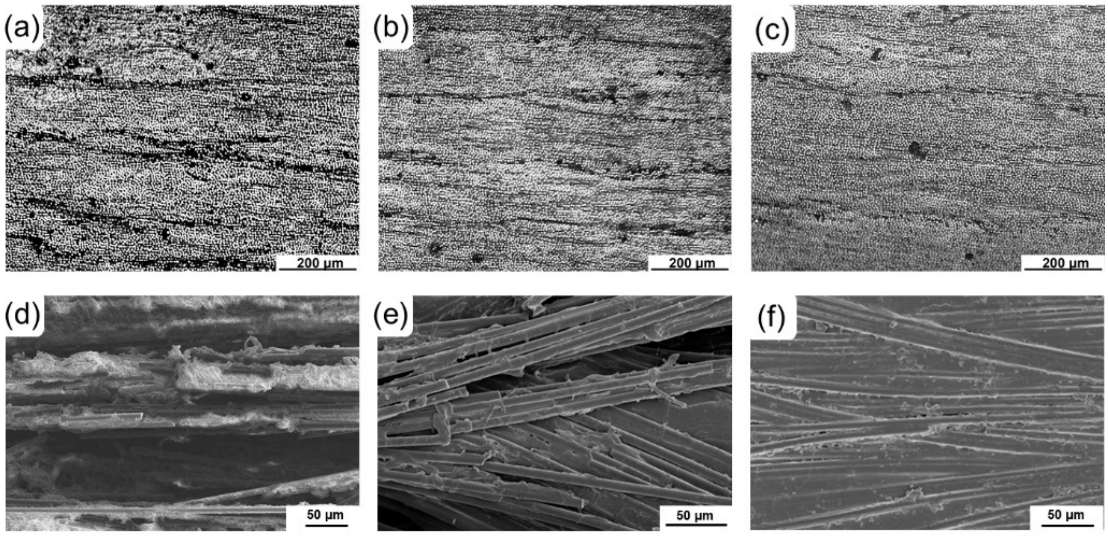

Figure S7. (a-c) The micrographs of the S1, S13 and C13 samples (from left to right); (d-f) the SEM images of the S1, S13 and C13 samples (from left to right).
